# Development and internal validation of a non-invasive clinical tool to predict sufficient omega-3 levels in early pregnancy

**DOI:** 10.1186/s12884-023-05687-2

**Published:** 2023-06-14

**Authors:** Joanna YX Fu, Carol A Wang, Ge Liu, Elyse Mead, Jason Phung, Maria Makrides, Craig E Pennell

**Affiliations:** 1 School of Medicine and Public Health, University of Newcastle, Callaghan, NSW 2300 Australia; 2 Hunter Medical Research Institute, New Lambton Heights, NSW 2305 Australia; 3 South Australian Health and Medical Research Institute, Adelaide, SA 5000 Australia; 4 John Hunter Hospital, New Lambton Heights, NSW 2305 Australia

**Keywords:** Omega-3 polyunsaturated fatty acids, n-3, Pregnancy, Prediction model, Development, Internal validation, Dietary questionnaire, Preterm birth

## Abstract

**Background:**

Complications from preterm birth (PTB) are the leading cause of death and disability in those under five years. Whilst the role of omega-3 (n-3) supplementation in reducing PTB is well-established, growing evidence suggests supplementation use in those replete may increase the risk of early PTB. Aim: To develop a non-invasive tool to identify individuals with total n-3 serum levels above 4.3% of total fatty acids in early pregnancy.

**Methods:**

We conducted a prospective observational study recruiting 331 participants from three clinical sites in Newcastle, Australia. Eligible participants (n = 307) had a singleton pregnancy between 8 and 20 weeks’ gestation at recruitment. Data on factors associated with n-3 serum levels were collected using an electronic questionnaire; these included estimated intake of n-3 (including food type, portion size, frequency of consumption), n-3 supplementation, and sociodemographic factors. The optimal cut-point of estimated n-3 intake that predicted mothers with total serum n-3 levels likely above 4.3% was developed using multivariate logistic regression, adjusting for maternal age, body mass index, socioeconomic status, and n-3 supplementation use. Total serum n-3 levels above 4.3% was selected as previous research has demonstrated that mothers with these levels are at increased risk of early PTB if they take additional n-3 supplementation during pregnancy. Models were evaluated using various performance metrics including sensitivity, specificity, area under receiver operator characteristic (AUROC) curve, true positive rate (TPR) at 10% false positive rate (FPR), Youden Index, Closest to (0,1) Criteria, Concordance Probability, and Index of Union. Internal validation was performed using 1000-bootstraps to generate 95% confidence intervals for performance metrics generated.

**Results:**

Of 307 eligible participants included for analysis, 58.6% had total n-3 serum levels above 4.3%. The optimal model had a moderate discriminative ability (AUROC 0.744, 95% CI 0.742–0.746) with 84.7% sensitivity, 54.7% specificity and 37.6% TPR at 10% FPR.

**Conclusions:**

Our non-invasive tool was a moderate predictor of pregnant women with total serum n-3 levels above 4.3%; however, its performance is not yet adequate for clinical use.

**Trial registration:**

This trial was approved by the Hunter New England Human Research Ethics Committee of the Hunter New England Local Health District (Reference 2020/ETH00498 on 07/05/2020 and 2020/ETH02881 on 08/12/2020).

**Supplementary Information:**

The online version contains supplementary material available at 10.1186/s12884-023-05687-2.

## Background

Preterm birth (PTB), defined as delivery before 37 completed weeks’ gestation, is the leading cause of death and disability in those under five years of age [1, 2]. Globally, PTB is estimated to affect 10.6% of births, equating to nearly fifteen million babies annually [2], and this rate is rising [2, 3]. The acute complications of PTB coupled with potential ongoing neurodevelopmental impairments have significant socioeconomic costs for families and health care systems [3]. Of babies born preterm, 20–30% are defined as early PTBs (i.e., delivery before 34 completed weeks’ gestation). This subgroup has the highest rates of morbidity and mortality [2, 4].

For most pregnancies, PTB is an idiopathic disorder with multifactorial causes, posing challenges for PTB prevention [5]. Current interventions with an established evidence base for most pregnancies include midwifery-led continuity care and screening for lower genital tract infections, with suggestive evidence for pharmacological smoking cessation to reduce rate of PTB [6].

The role of omega-3 (n-3) in the prevention of PTB has been studied for more than three decades in hundreds of thousands of women [7–9]. Omega-3s are polyunsaturated fatty acids (PUFAs) commonly obtained from marine, animal, and plant-based sources. After consumption, these essential fatty acids can be metabolised into more biologically potent lipid mediators known as long chain PUFAs (LC-PUFAs) [10]. The parent n-3 PUFA, α-linolenic acid (ALA) can be metabolised into eicosapentaenoic acid (EPA), docosapentaenoic acid (DPA) and docosahexaenoic acid (DHA) [10, 11] via alternating steps of desaturation and elongation by Δ6-desaturase and Δ5-desaturase, and elongase respectively [10, 12]. Whilst the exact mechanism(s) of action remains unclear, it has been proposed that increased dietary n-3 LCPUFAs may increase the production of n-3 prostaglandin derivatives which have less potent pro-inflammatory action compared to n-6 prostaglandin derivatives [13]. This interferes with the production of n-6 prostaglandin (E_2_ and F_2α_) which may play a role in premature uterine contractions and cervical ripening [14]. Further, possible anti-arrhythmic effects of n-3 on cardiac activity may translate to the myometrium, reducing electrical and contractile activity, ultimately delaying the onset of labour [15]. Overall, an individual’s n-3 level is associated with their dietary and supplementary intake of n-3 [16, 17] but may also be influenced by a number of non-dietary factors including age, body mass index (BMI), socioeconomic status (SES), smoking status, alcohol use, and presence of genetic variants involved in metabolism of PUFAs [16, 18, 19].

Studies evaluating the role of n-3 PUFA interventions on PTB rates have demonstrated mixed results. Taken together, the majority of studies suggest that n-3 supplementation may be valuable in PTB risk reduction [7–9], particularly in those with low levels of n-3 [20, 21]. Epidemiological evaluation of multiple large cohort studies has reported associations between increased n-3 intake and decreased PTB risk [22, 23]. Further, associations between decreased n-3 intake [24] or serum levels [25], and an increased risk of PTB have also been reported. Recent meta-analyses of more than 36 randomised controlled trials (RCTs) comparing n-3 supplementation versus controls or placebo have reported a 12% reduction in PTB (risk ratio (RR), 0.88; 95% CI (confidence intervals) 0.81–0.95). Regarding early PTB, meta-analyses have reported a 35% risk reduction (RR, 0.65; 95% CI 0.46–0.92) [9].

It has been proposed that the mixed results from the RCTs evaluating n-3 supplementation to prevent PTB may be due to variations in baseline n-3 levels in the populations studied [20, 26]. A recent cross-sectional analysis of n-3 intake in 184 countries demonstrated that increasing n-3 intake by one standard deviation in countries with low baseline n-3 intake (< 600 mg/day) was associated with a reduction in PTB [27]. In contrast, a similar increase in n-3 intake in countries with high baseline n-3 intake had no effect on PTB rate [27]. The results from the Omega-3 to Reduce the Incidence of Preterm Birth (ORIP) trial have taken this observation further; it reported that n-3 supplementation in those with low baseline levels of n-3 was associated with a 77% reduction in early PTB (RR, 0.23; 95%CI 0.07–0.79), whereas supplementation in those with high baseline levels of n-3 was associated with a more than two-fold increased risk of early PTB (RR, 2.27; 95%CI 1.13–4.58) [20, 28]. Taken together, these data highlight the potential importance of measuring serum n-3 levels prior to commencement of n-3 supplementation to reduce the rate of early PTB. Blood sampling for fatty acid analysis is an invasive procedure associated with pain, risk of infection and rarely, haemorrhage. This is currently the only way to accurately estimate baseline levels of n-3 at the start of pregnancy. It, however, is not part of routine antenatal screening and hence involves an expensive out-of-pocket cost, potentially precluding access for many patients for an important assessment. To our knowledge, no non-invasive tools have been developed to predict total n-3 serum levels above specific thresholds in early pregnancy. This resource gap highlights the need for the development of a non-invasive tool to determine baseline levels of n-3 early in pregnancy.

## Methods

The aim of this study was to develop and internally validate a non-invasive clinical tool to predict sufficient serum total n-3 levels (> 4.3% of total fatty acids) in early pregnancy. This study followed the Transparent Reporting of a multivariable prediction model for Individual Prognosis or Diagnosis (TRIPOD) checklist (See Additional file 1).

### Ethics

This observational study was approved by the Hunter New England Human Research Ethics Committee of the Hunter New England Local Health District (Reference 2020/ETH00498 and 2020/ETH02881). All participants provided informed written consent.

### Participants

A total of 331 participants were recruited in this prospective observational study from three sites (two pathology collection centres and one antenatal clinic) in Newcastle, Australia between March 2021, and March 2022. Participants were eligible if they had a singleton pregnancy between eight- and 20-weeks’ gestation at the time of recruitment. Pregnancy dating was calculated using a reliable menstrual period if available or confirmed by a first trimester ultrasound before 14 weeks gestation. If the two parameters differed by more than five days, dates from the ultrasound measurements were used. Participants were excluded if they had a multi-fetal pregnancy or did not provide complete dietary data or a blood sample.

### Data sources

For participants recruited at pathology collection centres, a blood sample was collected at recruitment. For participants recruited at the antenatal clinic, a blood sample was collected at their first trimester appointment. Participants ideally completed the questionnaire at the time of blood collection or where this was not possible due to time constraints, as soon as possible after blood collection.

Estimated n-3 intake was assessed using an electronic questionnaire based on the previously validated Australian Clinical Omega-3 Dietary Survey (CODS), a food frequency questionnaire used to assess intake of foods high in n-3 LFCPUFAs with consideration to serving size and frequency of consumption over the previous three months [29]. Servings sizes were estimated using a photo aid and/or a stated standardised portion size (i.e., one portion is approximately the size of a deck of cards). The frequency of consumption was quantified using open ended frequency categories (i.e., how many times per month, week, or day). Data on type, amount and frequency of n-3 supplementation use was also collected using this electronic questionnaire. Estimated weekly intake of n-3 PUFAs was quantified by conversion of questionnaire data using the Australian Food and Nutrient Database (AUSNUT 2011–2013) [30]. Estimated intake of n-3 PUFAs included ALA, EPA, DPA, DHA, total n-3 LCPUFAs (sum of EPA, DPA and DHA) and total n-3 PUFAs (sum of ALA, EPA, DPA and DHA) presented as milligrams per week (mg/week).

Maternal self-reported demographic data (including age, weight, height, residential postcode, highest level of completed education, multivitamin or supplementation use, smoking status, and alcohol intake) were collected using the electronic questionnaire. Body mass index was calculated by dividing the maternal weight (in kilograms) by height (in metres) squared. The maternal residential postcode was used to derive an Index of Relative Socioeconomic Advantage and Disadvantage (IRSAD). The index, ranged from 1 to 10 with a higher score indicating a relative lack of disadvantage and greater advantage, was a proxy for socioeconomic status.

Measured n-3 levels were evaluated from a single blood sample via venepuncture. Five millilitres (mL) of venous blood were collected in a serum separating gel tube. Samples were placed on ice for a maximum of four hours before being centrifuged at 3800 g for ten minutes at four degrees Celsius (°C). Serum samples were extracted, de-identified and stored at -80 °C until analysis. Fatty acid analysis was performed using gas chromatography at the South Australian Health and Medical Research Institute (SAHMRI) using methods published elsewhere [31].

### Outcomes

The primary outcome of this study was total serum n-3 PUFA levels above 4.3% of total fatty acids. This level was selected based on the dried blood spot data converted to a serum n-3 level using data supplied by SAHMRI [32].

### Statistical analysis

#### Model development

A full model approach was used for development of our prediction model. Factors that may influence serum n-3 levels were identified from the literature and were considered as candidate predictors; these included dietary and supplementary n-3 intake, maternal age, BMI, SES, smoking status, and alcohol use [16, 18, 19].

A proportion of ALA may be metabolised into n-3 LCPUFAs including EPA, DHA and DPA; however, the specific rate of conversion has not been well-established in the literature. Our prediction models accounted for conversions between 0 and 25% at 2.5% increments based on the evidence available [33]. All analyses performed were based on complete case analysis for dietary data. The association between predictors and the primary outcome was analysed by univariate regression models. Multivariate logistic regression, adjusting for variables including maternal age, BMI, IRSAD, n-3 supplementation use was used to develop multiple prediction models for measured n-3 levels. Smoking status and alcohol use were not included as there were too few events for these predictors for analysis.

#### Model internal validation

Internal validation of models was performed using 1000 bootstraps to generate 95% confidence intervals for performance metrics reported.

#### Model performance

Model performance was assessed by reporting sensitivity, specificity, accuracy, area under the receiver operating characteristic (AUROC) curve, maximum (Emax) and average (Eavg) difference in predicted versus less-calibrated probabilities, net reclassification improvement (NRI), Brier score, and scaled Brier score. Net benefit (NB) at threshold 50%, true positive rate (TPR) at 10% false positive rate (FPR) and TPR at 10% false negative rate (FNR), positive and negative predictive values (PPV and NPV) were evaluated to contextualise the model’s clinical value. Additionally, metrics that examined the model’s balance between specificity and sensitivity were also assessed. These metrics included Youden’s Index, Concordance Probability, Closest to (0,1) Corner and Index of Union [34].

#### Power analysis

Based on the ORIP data, we assumed the incidence of total serum n-3 levels above 4.3% would be 55% of the study population [20, 28] which gives a maximum R^2^_CS_ (max Cox-Snell R squared statistic), a pseudo measure of variance explained for models developed using logistic regression, of 0.75. Parameters for sample size calculation included adjusting for five predictors and using a conservative signal to noise ratio to give an adjR^2^_CS_ = 0.2*maxR^2^_CS_ [35, 36]). A subsequent step was also applied ensure the estimated sample size would not result in a model that would exceed the pre-specified shrinkage factor (s) of 0.90 and to ensure a small absolute difference of less than 0.05 between the developed model’s maxR^2^_CS_ and adjR^2^_CS_. A minimum of 276 women will be required to minimise variability in the prediction model to limit shrinkage to less than 10%.

All analyses were performed in R (v4.1.0) and its associate libraries [37].

## Results

### Participants

**Fig. 1:**
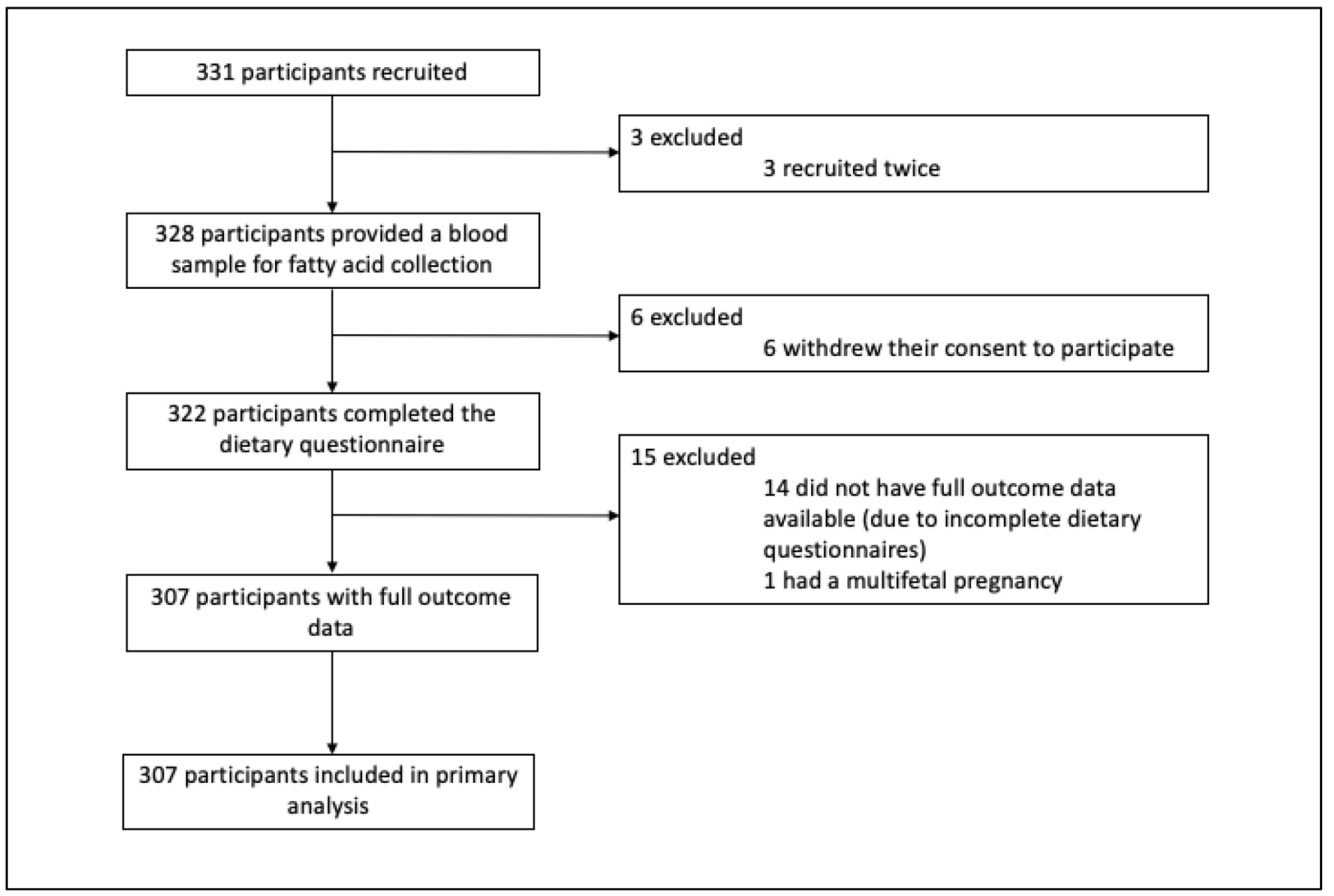
Participant flow through study

A total of 331 participants were recruited from three clinical sites in Newcastle. After withdrawal of consent, and exclusion due to incomplete dietary data, outcome data was available for 307 participants (92.7%) (Fig. 1).

Baseline maternal characteristics of the total cohort are as summarised in Table 1. The median maternal age in study participants was 32.5 years (interquartile range [IQR] 29.5–35.0). Most participants were Caucasian (90.7%) and the median BMI was 24.4 kg/m^2^ (IQR 22.2–29.0). The median gestational age at sample collection was 14.1weeks (IQR 11.8–15.3). Most study participants consumed pregnancy multivitamin supplements (n = 271, 88.3%) but only a small proportion took separate n-3 supplements (n = 18, 5.9%). Supplementation profiles of study participants are summarised in Table 2.

**Table 1:** Maternal baseline characteristics. All data are shown as median (IQR) or as number/total number (%)

Maternal Characteristics	Total Cohort (n = 307)	Cohort with n-3 serum levels > 4.3% (n = 180)	Cohort with n-3 serum levels ≤ 4.3% (n = 127)
Age, years	32.5 (29.5–35.0)	33.78 (30.2–35.1)	31.61 (28.8–34.8)
Weight, kg	69.0 (61.0–80.0)	68.00 (61.0–78.0)	70.0 (61.0–80.5)
BMI, kg/m^2^	24.4 (22.2–29.0)	24.28 (22.1–28.3)	24.49 (22.5–29.4)
Caucasian race	220/226 (90.7)	133/134 (99.3)	84/92 (91.3)
Completed high school education^†^	264/288 (91.7)	152/168 (90.5)	112/120 (93.3)
IRSAD score	5 (4.0–6.0)	5 (4.0–6.0)	5 (4.0–6.5)
Any tobacco use at study entry	5/305 (1.6)	2/178 (1.1)	3/127 (2.4)
Any alcohol use at study entry	9/304 (3.0)	7/177 (4.0)	2/127 (1.6)
Gestational age at blood collection, weeks	14.1 (11.8–15.3)	14.1 (11.8–15.1)	14.1 (11.8–15.6)
Primiparous	113/307 (36.8)	67/180 (37.2)	46/127 (36.2)
Previous preterm delivery	14/269 (5.2)	7/159 (4.4)	7/110 (6.4)
Consumed dietary supplements containing n-3 PUFAs at study entry	100/307 (32.6)	84/180 (46.7)	16/127 (12.6)

^†^Defined as completion of year 12

*IQR = interquartile range; BMI = body mass index, IRSAD = Index of Relative Socioeconomic Advantage and Disadvantage; n-3 = omega-3; PUFA = polyunsaturated fatty acid*

**Table 2:** Supplementation status in total cohort (n = 307). All data are shown as number (%)

	Supplementation Use					
**Serum total n-3 PUFA level (% of total fatty acids)**	No separate n-3 supplementation			Separate n-3 supplementation		
	No pregnancy multivitamin	Pregnancy multivitamin without n-3	Pregnancy multivitamin containing n-3	No pregnancy multivitamin	Pregnancy multivitamin without n-3	Pregnancy multivitamin containing n-3
**≤ 4.3**	19 (6.2)	92 (30.0)	13 (4.2)	1 (0.3)	2 (0.7)	0 (0.0)
**> 4.3**	16 (5.2)	80 (26.1)	69 (22.5)	0 (0.0)	11 (3.6)	4 (1.3)

n-3 = omega-3; PUFA = polyunsaturated fatty acid

Based on the modified CODS questionnaire, the median estimated weekly intake of total n-3 PUFAs was 4648.0 mg (IQR 6443.0). The median estimated weekly intake of ALA, and total combined DHA and EPA was 2487.0 mg and 1431.0 mg respectively. These intakes were significantly below the Australian National Heart Foundation dietary guidelines which recommend a daily intake of 1000 mg of ALA and 250-500 mg of DHA and/or EPA. This equates to a weekly intake of 7000 mg of ALA and 1750-3500 mg of DHA and/or EPA. Table 3 summarises the estimated weekly intake of n-3 PUFAs. The measured serum levels of n-3 PUFAs are also presented in Table 3.

**Table 3:** Estimated and measured levels of n-3 PUFAs. All data are shown as median (IQR).

n-3 PUFA Levels of Total Cohort (n = 307)	
ALA	
Estimated intake, mg/week	2487.0 (1141.0–5889.0)
Measured serum levels, % of total fatty acids	0.74 (0.61–0.89)
**EPA**	
Estimated intake, mg/week	351.6 (127.5–832.2)
Measured serum levels, % of total fatty acids	0.60 (0.47–0.75)
**DHA**	
Estimated intake, mg/week	820.0 (224.4–1457.7)
Measured serum levels, % of total fatty acids	2.57 (2.16–2.98)
**DPA**	
Estimated intake, mg/week	306.6 (170.2-459.3)
Measured serum levels, % of total fatty acids	0.50 (0.43–0.62)
**Total long-chain n-3 PUFAs** ^*^	
Estimated intake, mg/week	1431.0 (630.1–2682.9)
Measured serum levels, % of total fatty acids	3.73 (3.14–4.19)
**Total n-3 PUFAs** ^**†**^	
Estimated intake, mg/week	4648.0 (2285.0–8728.0)
Measured serum levels, % of total fatty acids	4.47 (3.96–4.96)

Estimated intake accounts for dietary and supplementary sources of n-3

^*^Total long-chain n-3 PUFAs are calculated from the sum of EPA, DHA and DPA

^†^Total n-3 PUFAs are calculated from the sum of ALA, EPA, DHA and DPA

*n-3 = omega-3; PUFA = polyunsaturated n-3 fatty acid; ALA = alpha-linolenic acid, EPA = eicosapentaenoic acid; DHA = docosahexaenoic acid; DPA = docosapentaenoic acid*

### Outcome

Of 307 participants included in the model development, 180 (58.6%) had a total n-3 serum level above 4.3% of total fatty acids.

### Model development

A total of 1370 models were examined. Predictors in the optimal model included dietary intake of total n-3 LCPUFAs (with no conversion of ALA to n-3 LCPUFAs), maternal age, BMI, IRSAD, and n-3 supplementation. The univariate analyses between predictors in the optimal model and outcome are presented in Table 4. Regression coefficients for predictors in the optimal prediction model are presented in Table 5.

**Table 4:** Predictors included in the optimal prediction model

Predictor	Regression Coefficients (β)	Standard Error	P value
Intercept	1.43	1.32	2.79 × 10^− 1^
Maternal age	-0.03	0.03	4.09 × 10^− 1^
BMI	0.01	0.02	5.93 × 10^− 1^
IRSAD	0.03	0.07	6.83 × 10^− 1^
Total n-3 LCPUFA intake	-1.51	0.34	1.12 × 10^− 5^
n-3 additions	-1.18	0.34	6.80 × 10^− 4^

*BMI = body mass index; IRSAD = Index of Relative Socioeconomic Advantage and Disadvantage; n-3 = omega-3; ALA = alpha-linolenic acid; PUFA = polyunsaturated fatty acid*

**Table 5:** Univariate analyses between predictors and outcome

Predictor	Regression Coefficients (β)	Standard Error	P value
Maternal age	-0.05	0.03	0.08
BMI	0.02	0.02	0.45
IRSAD	-0.04	0.06	0.53
Total n-3 LCPUFA intake	-2.01	0.29	3.92 × 10^− 11^
n-3 additions	-1.80	0.31	9.81 × 10^− 9^

*BMI = body mass index; IRSAD = Index of Relative Socioeconomic Advantage and Disadvantage; n-3 = omega-3; ALA = alpha-linolenic acid; PUFA = polyunsaturated fatty acid*

### Model performance

To optimise clinical relevance of identifying pregnant women with total serum n-3 levels above 4.3%, the top five models were selected by maximisation of sensitivity. The top five models are presented in Tables 6, 7, and 8. From these top five models, the optimal model was selected with consideration to the Youden’s Index, Concordance Probability, Closest to (0,1) Corner and Index of Union (Table 8). Further, the assessment of metrics in additional domains including overall performance (Brier Score, Scaled Brier Score in Table 6), calibration (Emax, Eavg in Table 6), NRI (Table 6) and NB (Table 7) demonstrated consistency in the predictive performance of optimal model selected for this study.

**Table 6:** Overall performance measures (95% confidence interval) for top five models

Model Predictors^*^	Estimated n-3 intake (mg/week)	AUROC	Accuracy	Sensitivity	Specificity	Emax	Eavg	NRI	Brier’s Score	Scaled Brier’s Score
Dietary and supplementary intake of total n-3	767.8	0.744 (0.742–0.746)	0.722 (0.720–0.724)	0.847 (0.844–0.850)	0.547 (0.543–0.551)	0.160 (0.157–0.164)	0.072 (0.070–0.073)	0.197 (0.195–0.199)	0.200 (0.199–0.367)	0.170 (0.165–0.175)
Dietary and supplementary intake of total n-3 with 1.0% ALA conversion	797.0	0.741 (0.738–0.743)	0.714 (0.712–0.716)	0.837 (0.834–0.840)	0.544 (0.539–0.548)	0.160 (0.156–0.164)	0.071 (0.069–0.073)	0.190 (0.188–0.192)	0.202 (0.201–0.368)	0.162 (0.157–0.203)
Dietary and supplementary intake of total n-3 LCPUFA	630.1	0.742 (0.739–0.744)	0.710 (0.708–0.712)	0.870 (0.866–0.874)	0.488 (0.483–0.492)	0.163 (0.159–0.167)	0.072 (0.071–0.074)	0.179 (0.177–0.181)	0.200 (0.199–0.370)	0.173 (0.167–0.176)
Dietary and supplementary intake of total n-3 with 1.0% ALA conversion	670.9	0.733 (0.731–0.736)	0.694 (0.692–0.696)	0.845 (0.840–0.849)	0.484 (0.479–0.489)	0.160 (0.156–0.164)	0.072 (0.070–0.074)	0.164 (0.162–0.166)	0.204 (0.203–0.373)	0.153 (0.148–0.157)
Dietary and supplementary intake of total n-3 LCPUFA	558.1	0.732 (0.729–0.734)	0.689 (0.687–0.691)	0.851 (0.846–0.856)	0.465 (0.460–0.471)	0.164 (0.160–0.168)	0.073 (0.071–0.075)	0.158 (0.156–0.160)	0.204 (0.203–0.373)	0.154 (0.149–0.158)

^*^All models were adjusted for maternal age, BMI, IRSAD and n-3 additions. Dietary intake refers to n-3 from food items whilst supplementary intake refers to n-3 from supplement use

*Mg = milligrams; AUROC = area under the receiver operating characteristic; NRI = net reclassification improvement; n-3 = omega-3; ALA = alpha-linolenic acid*

**Table 7:** Performance measures (95% confidence interval) assessing clinical utility

Model Predictors^*^	Estimated n-3 intake (mg/week)	NB	TPR at 10% FPR	TNR at 10% FNR	PPV	NPV
Dietary and supplementary intake of total n-3	767.8	0.306 (0.303–0.310)	0.376 (0.369–0.383)	0.360 (0.353–0.367)	0.725 (0.723–0.728)	0.719 (0.715–0.724)
Dietary and supplementary intake of total n-3 with 1.0% ALA conversion	797.0	0.299 (0.296–0.302)	0.359 (0.352–0.365)	0.361 (0.355–0.368)	0.721 (0.719–0.724)	0.706 (0.701–0.710)
Dietary and supplementary intake of total n-3 LCPUFA	630.1	0.295 (0.292–0.298)	0.420 (0.414–0.426)	0.363 (0.357–0.370)	0.706 (0.703–0.708)	0.734 (0.728–0.739)
Dietary and supplementary intake of total n-3 with 1.0% ALA conversion	670.9	0.278 (0.275–0.282)	0.381 (0.375–0.387)	0.367 (0.360–0.373)	0.698 (0.696–0.701)	0.697 (0.692–0.702)
Dietary and supplementary intake of total n-3 LCPUFA	558.1	0.274 (0.271–0.277)	0.396 (0.391–0.401)	0.367 (0.360–0.373)	0.693 (0.690–0.695)	0.701 (0.695–0.706)

^*^All models were adjusted for maternal age, BMI, IRSAD and n-3 additions. Dietary intake refers to n-3 from food items whilst supplementary intake refers to n-3 from supplement use

*Mg = milligrams; NB = net benefit; TPR = true positive rate; FPR = false positive rate; TNR = true negative rate; FNR = false negative rate; PPV = positive predictive value; NPV = false negative value; n-3 = omeg-3; LCPUFA = long-chain polyunsaturated fatty acids*

**Table 8:** Performance metrics (95% confidence interval) balancing sensitivity and specificity

Model Predictors^*^	Estimated n-3 intake (mg/week)	Youden’s Index	Concordance Probability	Closest to (0,1) Corner	Index of Union
Dietary and supplementary intake of total n-3	767.8	0.394 (0.390–0.398)	0.462 (0.459–0.465)	0.546 (0.542–0.550)	0.303 (0.298–0.309)
Dietary and supplementary intake of total n-3 with 1.0% ALA conversion	797.0	0.380 (0.376–0.385)	0.453 (0.450–0.457)	0.489 (0.486–0.493)	0.298 (0.293–0.304)
Dietary and supplementary intake of total n-3 LCPUFA	630.1	0.358 (0.353–0.362)	0.422 (0.419–0.425)	0.534 (0.530–0.537)	0.386 (0.380–0.393)
Dietary and supplementary intake of total n-3 with 1.0% ALA conversion	670.9	0.329 (0.325–0.333)	0.405 (0.402–0.409)	0.482 (0.478–0.486)	0.369 (0.362–0.376)
Dietary and supplementary intake of total n-3 LCPUFA	558.1	0.361 (0.312–0.320)	0.392 (0.388–0.395)	0.563 (0.559–0.567)	0.395 (0.387–0.403)

^*^All models were adjusted for maternal age, BMI, IRSAD and n-3 additions. Dietary intake refers to n-3 from food items whilst supplementary intake refers to n-3 from supplement use

*Mg = milligrams; n-3 = omega-3; LCPUFA = long-chain polyunsaturated fatty acids; ALA = alpha-linolenic acid*

**Fig. 2:**
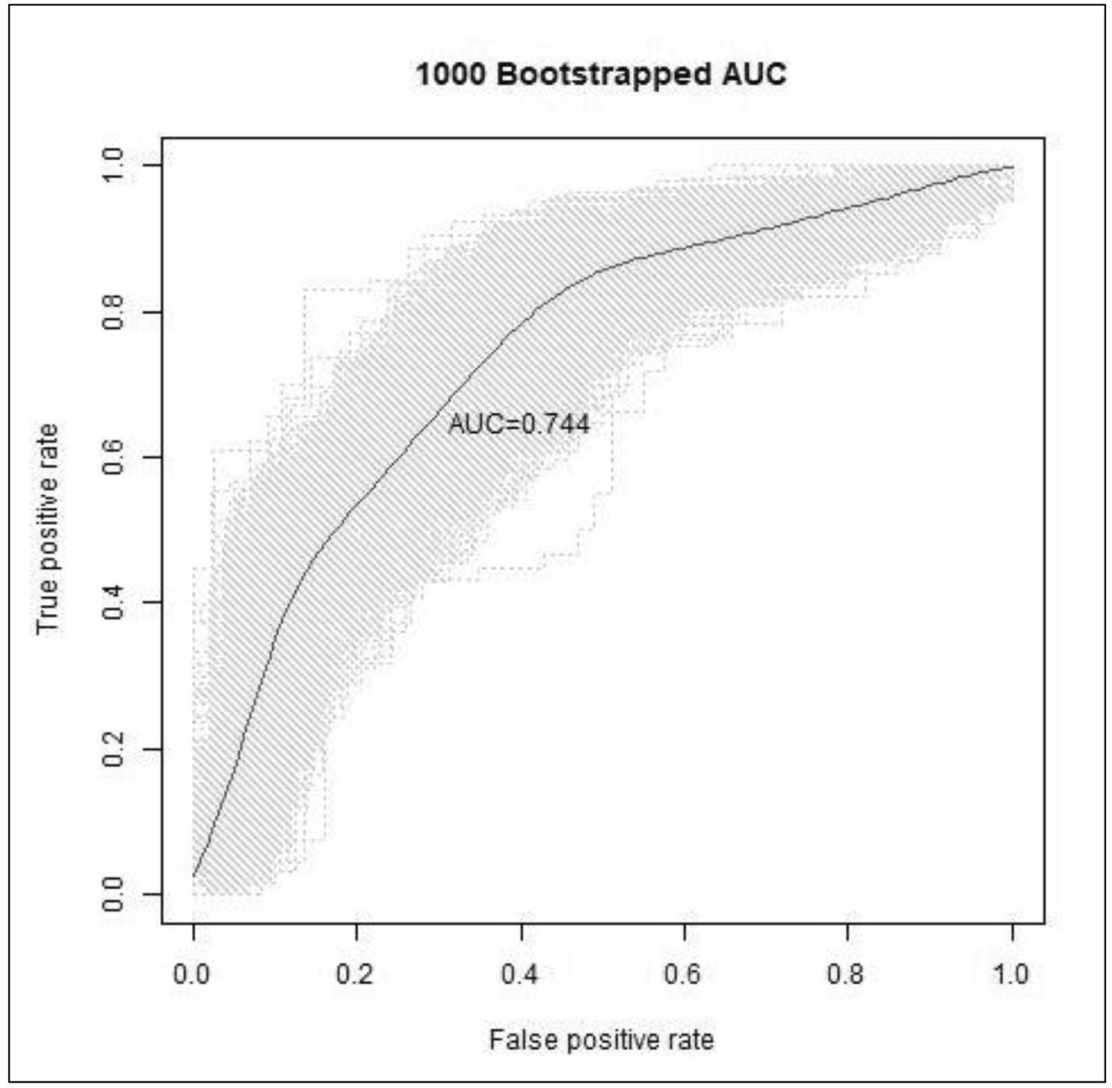
Receiver operating characteristic curve of the optimal prediction model n-3 = omega-3; AUC = area under the curve

**Fig. 3:**
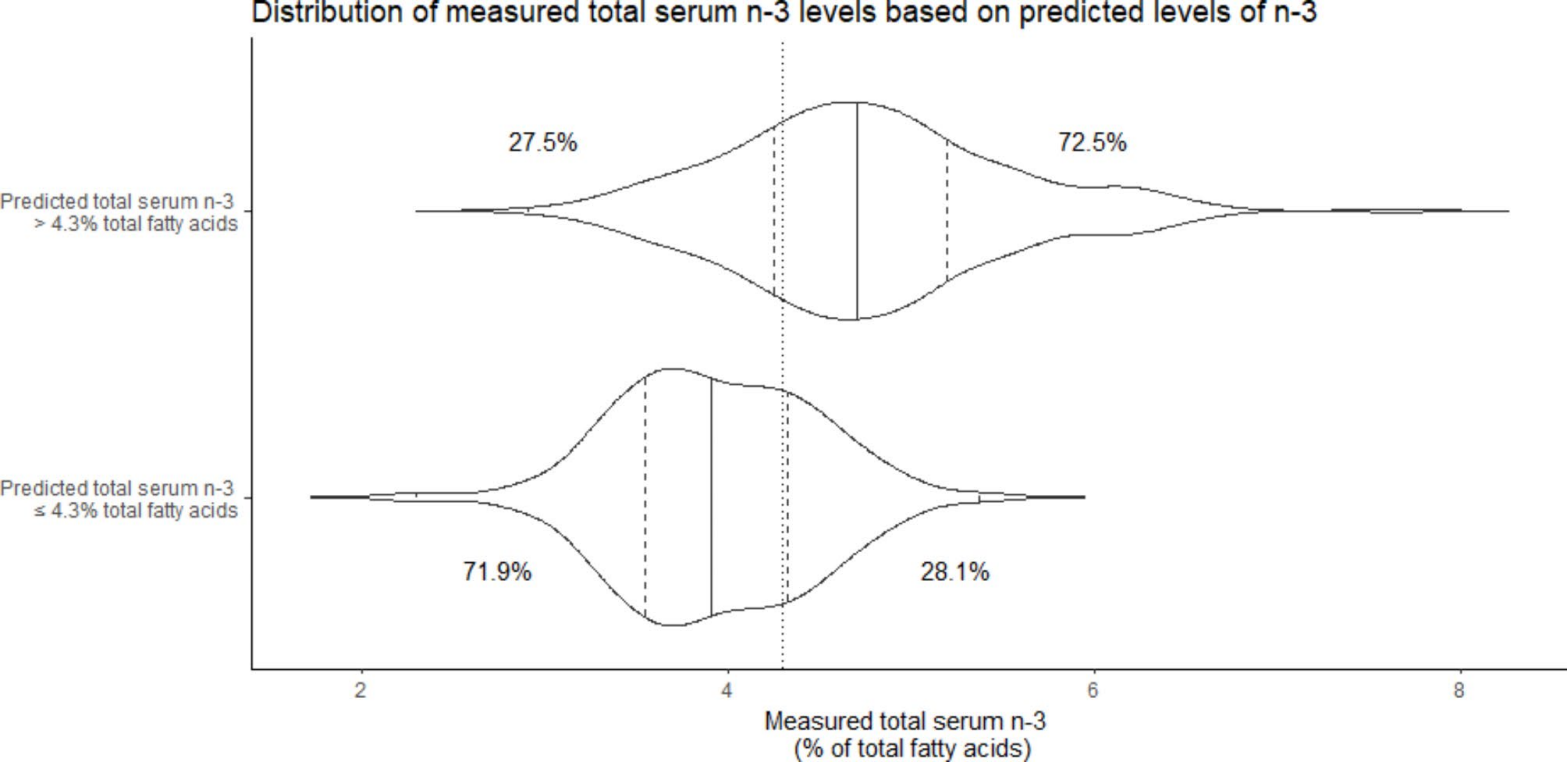
Distribution of measured total serum n-3 levels based on predicted levels of n-3 n*-3 = omega-3*

An estimated n-3 intake above 767.8 mg/week with adjustment for maternal sociodemographic factors was predicted to give a total serum n-3 level above 4.3%. The optimal prediction model had an AUROC of 0.744 (95% CI 0.742–0.746), a sensitivity of 84.7%, a specificity of 54.7%, TPR at 10% FPR of 37.6%, and TNR at 10% FNR of 36.0%. These data are presented graphically in Fig. 2. Distribution data for the measured n-3 levels for those predicted to be above and below 4.3% of total fatty acids are presented in Fig. 3. In those predicted to have measured n-3 levels greater than 4.3%, 72.5% had measured n-3 levels greater than 4.3% (i.e., the PPV was 72.5%). Conversely, in those predicted to have measured levels less than or equal to 4.3%, 71.9% had concordant measured n-3 levels (i.e., NPV was 71.9%).

## Discussion

In this study, we developed a non-invasive dietary prediction tool to identify a sub-population of pregnant women who are likely to have a total serum n-3 level above 4.3% of total fatty acids based on their dietary and demographic characteristics. This tool took an average of six minutes to administer and had moderate test performance with 84.7% sensitivity and 54.7% specificity. To our knowledge, this is the first study to develop a model to predict n-3 serum levels above or below a given threshold in pregnancy.

A model has previously been developed in a non-pregnant population that aimed to predict the effect of n-3 supplementation on erythrocyte EPA and DHA levels [38]. In contrast to our study, their primary outcome was a continuous measure. The non-pregnant model observed that EPA and DHA levels had a modest correlation with predicted EPA and DHA levels, with a R^2^ of 0.69 [38]. Similar to our study, their prediction model included the usage of n-3 supplementation. Other studies in pregnant populations have examined the associations between dietary and demographic factors on n-3 markers [39, 40], with negative associations detected between smoking status and alcohol use with n-3 levels [40]. We could not evaluate the role of smoking or alcohol use in this study as there were very few smokers or mothers consuming alcohol in our study population.

This study demonstrates the potential to predict pregnant women with total serum n-3 levels above 4.3% of total fatty acids using basic sociodemographic data and a short nutrition questionnaire. Using our optimal prediction model, we had a sensitivity of 84.7%. For clinical utility, a sensitivity of 90% would be optimal to minimise any potential harm from n-3 supplementation in pregnancy to women with high baseline n-3 levels. In general, an AUROC curve of 0.5 suggests no discrimination, 0.7 to 0.8 is considered acceptable, 0.8 to 0.9 is considered excellent, and more than 0.9 is considered outstanding [41]. The performance measures of our optimal model demonstrate acceptable discrimination, however, in its current form, our prediction model is not appropriate for routine antenatal care. Further, this tool has been developed to predict serum n-3 levels associated with an increased risk of early PTB with additional n-3 supplementation and consequently is not applicable to other perinatal outcomes including small for gestational age or intrauterine growth restriction, Currently, there are no well-defined serum n-3 levels predictive for other perinatal outcomes.

To improve model performance, adjustment for factors including alcohol use and smoking status may be useful [40]. Further, adjusting for total energy intake may be valuable as it has been shown to improve the correlation between estimated dietary intake and measured nutrient concentrations in blood [42]. However, additional assessment of total energy intake to our predictive model would substantially increase the time required to complete the questionnaire. This may be prohibitive when designing a tool to screen all pregnant women. Further, future prediction models may need to be more complex and capable of predicting more than one threshold. This need is highlighted by the recent South Australian recommendations based on ORIP trial data that recommends three specific n-3 supplementation guidelines dependent on baseline serum n-3 levels. Pregnant women with total serum n-3 levels below 3.7% of total fatty acids are recommended to begin n-3 supplementation, with evidence suggesting the potential to reduce the risk of early PTB by 77% [20]. For those with levels between 3.7% and 4.3%, no change in n-3 supplementation status is required. Women with levels above 4.3% who are taking high dose supplementation (> 1 g/day) are recommended to cease supplementation if it has already been commenced or refrain from initiating additional high dose n-3 supplementation (> 1 g/day) as it may increase the risk of early PTB by 2.27-fold [43]. Ideally, development of a non-invasive multinomial model to predict membership to one of the three groups (< 3.7%, 3.7 to 4.3% and > 4.3%) may be valuable in optimising which pregnant women should be offered n-3 supplementation. However, development of such a model presents significant statistical challenges where careful consideration is required in the rate of various events in each group and the number of predictors accounted for in the model [44]. Based on the distribution of women from this study and the ORIP trial, the rate of each event (i.e., total n-3 serum levels < 3.7%, 3.7–4.3% and > 4.3%) is estimated to be 15–20%, 20–40% and 55–60%, respectively. Assuming adjustment for up to eight predictors (i.e., dietary intake of n-3, supplementary n-3 use, total energy intake, maternal age, BMI, socioeconomic status, alcohol use and smoking status), an estimated 600–1200 participants will be required to ensure ten to twenty events occur per variable to perform a sufficiently powered study [44].

**Fig. 4:**
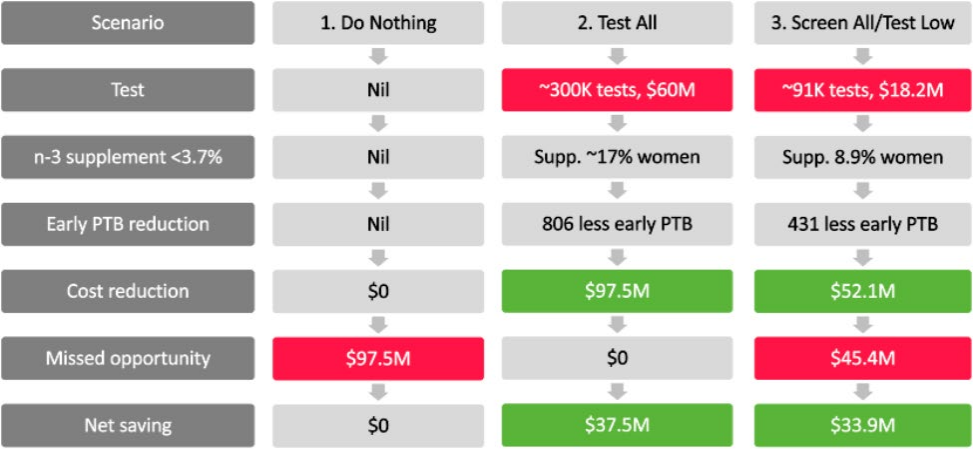
Scenarios for n-3 supplementation using screening and/or testing of maternal n-3 baseline levels *~ = approximately; M = million; n-3 = omega-3; supp. = supplementation*

One potential use for our prediction tool may be used to reduce the number of pregnant women requiring expensive serum testing (Fig. 4.) In this scenario, women with singleton pregnancies with predicted total n-3 serum levels equal to or below 4.3% of total fatty acids should undergo definitive serum fatty acid testing and receive n-3 supplementation as appropriate. In contrast, women with singleton pregnancies with predicted total n-3 serum levels above 4.3% of total fatty acids, do not undergo further serum fatty acid testing or n-3 supplementation. This reduces the test burden to 30% of our study population. Our predictive tool was able to correctly classify 72.7% of the population to this higher group. However, due to the limited predictive ability of our developed tool, 19% of the total study population were incorrectly classified and may miss out on potential benefits of n-3 supplementation. By utilising Australian birth data [45] and n-3 fatty acid results of Australian pregnant women [32], using the predictive tool as a screening tool to reduce serum testing, resulted in an estimated total saving of AUD$33.9 million when the financial burden of preterm birth was considered. In Fig. 4, this scenario (3. Screen all/test low) is compared to two other scenarios with consideration to the percentage of the population undergoing n-3 screening, testing and supplementation and the costs associated. In these scenarios, definitive serum fatty acid testing cost $200 which is congruent with costs in NSW [46].

Overall, this study has several strengths. First, the sample size is adequate to minimise variability in the prediction model to ideally minimise shrinkage to less than 10%. Second, the inclusion criteria for this study were broad increasing its generalisability to an Australian population. Third, we followed guidelines and recommendations for development of prediction models supported by current literature [47–50]. For example, internal validation was performed with bootstrapping to assess for potential overfitting and optimism. Bootstrapping is considered a superior approach compared to other methods as it provides stable estimates with less bias [51]. Fourth, the prediction tool developed was non-invasive, inexpensive, and quick to administer.

There are several important limitations that should be considered when interpreting this study. First, n-3 dietary intake was evaluated using a dietary questionnaire which has an inherent risk of recall bias and misreporting. Our modified questionnaire assessed three months of intake retrospectively which may increase recall bias. To minimise misreporting of food portions, we included visual aids where possible. Second, adjustment for total energy intake was not performed in this study which may attenuate the correlations between dietary intake and biomarkers of n-3. Third, other factors that may influence n-3 levels including physical activity levels, alcohol intake, smoking status and genetic differences between individuals were also not considered in our analysis. Fourth, no external validation has yet been performed for our optimal prediction model. Fifth, due to wide variation in population based nutritional profiles, the generalisability of this tool is likely to be limited to an Australian Caucasian population. Our dietary questionnaire assessed consumption of n-3 food items common to an Australian diet and the proportion of n-3 in food items was defined by an Australian national nutrient database. For applicability in other populations, this prediction tool may need to be customised to population-based nutritional profiles.

## Conclusion

In conclusion, we developed a simple tool to predict the subgroup of pregnant women who have total serum n-3 levels above 4.3% based on their dietary intake of n-3 and sociodemographic factors. Although our optimal prediction model only had moderate test performance, this study demonstrates the potential for inexpensive and non-invasive tools to identify baseline maternal n-3 status in early pregnancy which may play a valuable role in optimising n-3 supplementation. Before this tool can be applied to the clinical setting, further enhancement of model performance and model validation is required.

## Electronic supplementary material

Below is the link to the electronic supplementary material.

**Additional file 1. Supplementary Table 1**. Transparent Reporting of Individual Prognosis and Diagnosis (TRIPOD) Checklist

## Data Availability

The datasets used and/or analysed during the current study are available from the corresponding author on reasonable request.

## Abbreviations

ALA: α-linolenic acid
AUROC: Area under the receiver operating characteristic
CI: Confidence interval
DHA: Docosahexaenoic acid
DPA: Docosapentaenoic acid
EPA: Eicosapentaenoic acid
FNR: False negative rate
FPR: False positive rate
LCPUFA: Long chain polyunsaturated fatty acid
n-3: Omega-3
NB: Net benefit
NPV: Negative predictive value
NRI: Net reclassification improvement
ORIP: Omega-3 to Reduce the Incidence of Preterm Birth
PPV: Positive predictive value
PTB: Preterm birth
PUFA: Polyunsaturated fatty acid
RCT: Randomised controlled trial
RR: Risk ratio
TRIPOD: Transparent Reporting of a multivariable prediction model for Individual Prognosis or Diagnosis
TPR: True positive rate
TNR: True negative rate
